# Effect of continuous high-volume hemofiltration on patients with acute respiratory distress syndrome

**DOI:** 10.1186/cc12369

**Published:** 2013-03-19

**Authors:** A Kuzovlev, E Tishkov, O Bukaev

**Affiliations:** 1 V.A. Negovsky Scientific Research Insitute of General Reanimatology RAMS, Moscow, Russia; 2 A.I. Evdokimov Moscow State University of Medicine and Dentistry, Moscow, Russia

## Introduction

The aim of this study was to investigate the effects of intermittent venovenous high-volume hemofiltration (IHVHF) on extravascular lung water, respiratory function and cytokine removal in patients with acute respiratory distress syndrome (ARDS) associated with postoperative multiple organ dysfunction syndrome in severe abdominal infection patients.

## Methods

Thirty patients with severe abdominal infection and ARDS were randomized into the IHVHF group (*n *= 15) and control group (*n *= 15). All patients were mechanically ventilated. The IHVHF technique with a rate of 100 to 1203 ml/kg/hour during 6 to 8 hours was used. The PiCCOplus system was used to monitor cardiac output (CO), extravascular lung water index (EVLWI) and intrathoracic blood volume index (ITBVI). Arterial partial pressure of oxygen (PaO_2_), arterial partial pressure of carbon dioxide (PaCO_2_), oxygenation index (PaO_2_/FiO_2_) and dynamic lung compliance (Cdyn), oxygen delivery and oxygen consumption were measured. Serum concentrations of TNFa, IL-6, IL-8 and lactate were measured by ELISA method. The APACHE II scores before and after IHVHF therapy were detected.

## Results

All indexes in the control group did not show any significant improvement before and after treatment (*P *0.05). There was a significant decrease of serum concentrations of TNFa, IL-6, IL-8 and lactate, increase of CO and decrease of EVLWI 24 hours after IHVHF (*P *0.05). The indexes of oxygen delivery, consumption, PaO_2_, and Cdyn improved significantly, PaO_2_/FiO_2 _increased markedly compared with those before IHVHF and the control group. Significant differences were detected in all of the hemodynamics and oxygen indexes (*P *0.05).

## Conclusion

IHVHF presents with a significant beneficial effect on respiratory function in patients with ARDS as a result of removal of cytokines, decrease in EVLWI, improvement of hemodynamic parameters, and correction of disturbances in oxygen balance. IHVHF adjuvant treatment for ARDS reduces pulmonary edema, improves PaO_2_/FiO_2 _and Cdyn and mechanical ventilation parameters. IHVHF may be a promising treatment for ARDS in postoperative multiple organ dysfunction syndrome in severe abdominal infection patients.
